# Effect of supplemental glycerol monolaurate and oregano essential oil blend on the growth performance, intestinal morphology, and amino acid digestibility of broiler chickens

**DOI:** 10.1186/s12917-021-03022-5

**Published:** 2021-09-25

**Authors:** Shimaa A. Amer, Samar A. Tolba, Dina M. M. AlSadek, Doaa M. Abdel Fattah, Aziza M. Hassan, Abdallah E. Metwally

**Affiliations:** 1 Department of Nutrition and Clinical Nutrition, Faculty of Veterinary Medicine, Zagazig University, Zagazig, 44511 Egypt; 2 Department of Histology and Cytology, Faculty of Veterinary Medicine, Zagazig University, Zagazig, 44511 Egypt; 3 Department of Biochemistry, Faculty of Veterinary Medicine, Zagazig University, Zagazig, 44511 Egypt; 4 Department of Biotechnology, College of Science, Taif University, P.O. Box 11099, Taif, 21944 Saudi Arabia

**Keywords:** Broiler chickens, Glycerol monolaurate, Oregano essential oil, Growth performance, Intestinal histomorphology, Ileal digestibility

## Abstract

**Background:**

This experiment tested the impact of the combined supplementation of glycerol monolaurate (GLM) and oregano essential oil (EO) to broiler diets. Growth performance, metabolic response, immune status, apparent ileal digestibility coefficient (AID%), and intestinal histomorphology were assessed. Three-day-old Ross-308 broilers (76.62 g ± 0.50*, n* = 240) were randomly allocated into 4 experimental groups (6 replicates/group and 10 chicks/replicate). Birds were fed corn-soybean meal basal diets supplemented with four levels of GLM and oregano EO blend: 0, 0.15, 0.45, and 0.75% for 35 days.

**Results:**

During the starter period, dietary GLM and oregano EO did not show significant (*P >* 0.05) changes in growth performance. During the grower period, GLM and oregano EO supplemented groups showed a linear and quadratic decline in FCR. During the finisher and overall performance, a linear increase in the body weight (BW), body weight gain (BWG), the protein efficiency ratio (PER), and relative growth rate (RGR), and a linear decrease in the FCR at 0.75% dietary level of GLM and oregano EO compared to the control. The broken-line regression model showed that the optimum dietary level of GLM and oregano EO blend was 0.58% based on final BW and FCR. The 0.45% or 0.15% dietary level of supplemented additives lowered (*P* < 0.05) the AID% of threonine and arginine, respectively, with no change in the AID% of other assessed amino acids at all dietary levels. Muscle thickness in jejunum and ileum in all dietary supplemented groups was increased (*P* < 0.05); however, such increase (*P* < 0.05) in the duodenum was shown at 0.45 and 0.75% dietary levels. All GLM and oregano EO supplemented groups showed increased (*P* < 0.05) duodenal, jejunal, and ileal villus height. The 0.15 and/or 0.75% dietary levels of supplemented additives increased (*P* < 0.05) the ileal and duodenal crypt depth, respectively, with a decreased (*P* < 0.05) duodenal crypt depth at 0.15% dietary level. The goblet cell count in ileum decreased (*P* < 0.05) in all GLM and oregano EO supplemented groups, but this decreased count (*P* < 0.05) was detected in jejunum at 0.45 and 0.75% dietary levels. The GLM and oregano EO supplemented groups did not show significant (*P >* 0.05) changes in the assessed metabolic and immune status parameters. Economically, the total return and performance index was increased at 0.75% dietary level.

**Conclusion:**

Better growth performance was achieved at a 0.75 % dietary level of GLM and oregano EO by improving most intestinal morphometric measures. The optimum dietary level detected was 0.58%. The lack of influence of supplemented additives on chickens' immune and metabolic responses could indicate a lack of synergy between GLM and oregano EO.

## Background

Feed contributes 65-70% of the overall poultry production cost, and economic poultry production primarily depends on efficient feed utilization to increase chickens’ performance and overall productivity [1]. However, there is criticism on using chemical feed additives as growth promoters in poultry feed due to harmful effects on consumers and increased demand for organic poultry production. Moreover, more pressure has been placed on researchers and livestock farmers to use antibiotics as growth promoters in livestock feed since the European Union's ban [2]. Thus, the poultry production challenge uses natural alternatives with comparable beneficial impacts to antibiotics and chemical feed additives to boost poultry health and growth performance. Accordingly, biologically active materials are gaining more attention in the current post-antibiotic era as alternative growth promoters in the poultry diet [3–5]. Among these materials are medium-chain fatty acids (MCFAs).

Medium-chain fatty acids are a group of FAs with 6 to 12 carbon atoms derived by lipid fraction separation from edible fats such as milk fat and coconut oil, which are namely caproic (C6:0), caprylic (C8:0), capric (C10:0), or lauric acid (C12:0) [6]. The advantageous use of MCFAs over long-chain fatty acids (LCFAs) in poultry feed is due to differences in the energy ratio between MCFAs and LCFAs, which can influence the energy balance inside the body because of the different effects on intake, transport, and utilization efficiency [7]. Medium-chain α-monoglycerides (MG) are promising feed supplements for broilers production. Glycerol monolaurate (GML) is a chemical compound made from lauric acid and glycerol that exhibits strong antimicrobial activity [8]. In a previous report, we investigated the influence of glycerol monolaurate (GLM) as MCFA on the broilers’ performance that showed an enhancement in the birds’ immune status and intestinal histomorphology without affecting the growth performance or ileal amino acid (AA) digestibility [9]. To the best of our knowledge, few studies have demonstrated the combined effect of MCFAs with other nutrients in the poultry diet. Omar *et al.* [10] assessed the potential effects of using a combination of MCFAs and thymol oil (0.1g EOs/kg diet + 0.1% MCFAs); they found the best FCR economic value during the period from 7-38 days. Published reports proposed phytogenic feed additives as a promising natural source of bioactive metabolites in poultry feed, promoting growth performance and general health of poultry [11, 12].

Oregano (*Origanum spp.)* is an aromatic herb that contains plentiful active components that have been employed in poultry feed to replace chemical antibiotics [13]. The essential oil from oregano obtained by the steam-distillation process from leaves and flowers is known for its antioxidative activity [14]. Terpenoid compounds such as thymol and carvacrol are the main components of oregano EO that have shown positive effects on broiler growth performance, intestinal morphology, and immune responses, partly attributed to their antioxidant and antimicrobial activities [15, 16]. However, to date, the exact mode of action of phytogenic feed additives remains scientifically interesting; Given its long history of use, it is still not clear. Therefore, a broiler chicken experiment was performed to assess the effectiveness of the combined inclusion of different dietary levels of GLM and oregano EO. We tested their effects on the broilers’ growth performance, metabolic status, immune status, ileal AA digestibility, and intestinal histomorphology to justify the impacts of such additives.

## Results

### Growth performance

**Fig. 1:**
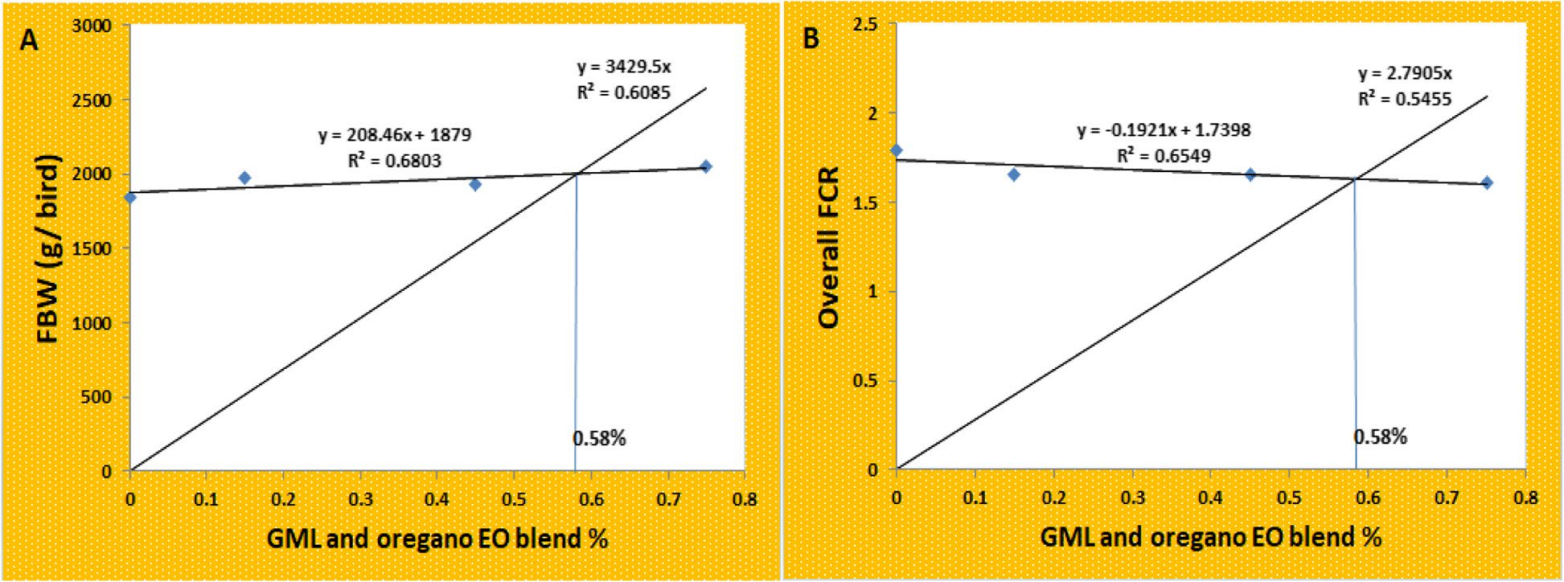
Broken-line regression model showing the optimum dietary level of GML and oregano EO using the data of final body weight (FBW) (**A**) and overall FCR (**B**)

Growth performance findings are listed in Table 1. Throughout the starter period (4-10 d), dietary GLM and oregano EO did not result in significant (*P >* 0.05) changes in BW, BWG, FI, or FCR. During the grower (11-22 d), GLM and oregano EO supplemented groups showed a linear and quadratic decline (*P* < 0.05) in FCR, compared to the control; however BW, BWG, and FI did not show a significant (*P >* 0.05) change. For the finisher period (23-35 d), the dietary GLM and oregano EO at the level of 0.75% led to a linear increase in the BW and BWG and a linear decrease (*P* < 0.05) in FCR compared to the control group; nevertheless, the feed intake did not show a significant (*P >* 0.05) alteration. The overall performance showed a linear increase in the BW, BWG, PER, and RGR and a linear decrease in the FCR (*P* < 0.05) at a 0.75% dietary level of GLM and oregano EO compared to the control. The optimum dietary level of GLM and oregano EO blend was 0.58% based on final BW and FCR (Fig. 1).

**Table 1:** Effects of supplemental GLM and oregano EO blend on the broilers’ growth performance

Parameters	T1	T2	T3	T4	SEM	Regression *		
						Linear	Quadratic	Cubic
Initial Wt. (g)	75.66	76.58	76.83	76.83	0.26	0.32	0.56	0.90
Starter period								
BW(g)	217.55	224.75	216.91	211.79	2.76	0.51	0.46	0.62
BWG(g)	141.88	148.16	140.08	134.95	2.61	0.42	0.46	0.60
FI (g)	186.55	191.75	183.50	176.95	2.45	0.26	0.41	0.62
FCR	1.31	1.29	1.31	1.31	0.01	0.86	0.75	0.69
Grower period								
BW(g)	836.88	893.87	855.58	895.41	11.64	0.38	0.80	0.25
BWG(g)	619.33	669.12	638.66	683.62	9.73	0.20	0.92	0.20
FI (g)	976.11	917.95	885.33	943.45	16.59	0.56	0.24	0.57
FCR	1.57^a^	1.37^b^	1.38^b^	1.37^b^	0.02	0.008	0.03	0.20
Finisher period								
BW(g)	1844.22^b^	1973.08^ab^	1933.95^ab^	2046.04^a^	21.85	0.04	0.87	0.19
BWG(g)	1007.33^b^	1079.20^ab^	1078.37^ab^	1150.62^a^	15.08	0.02	0.99	0.37
FI(g)	2014.00	2024.16	1997.50	2061.20	19.57	0.68	0.65	0.62
FCR	1.99^a^	1.88^ab^	1.85^ab^	1.79^b^	0.01	0.01	0.60	0.56
Overall performance								
BW(g)	1844.22^b^	1973.08^ab^	1933.95^ab^	2046.04^a^	1844.22^b^	0.04	0.87	0.19
BWG(g)	1768.55^b^	1896.50^ab^	1857.12^ab^	1969.20^a^	21.66	0.04	0.88	0.18
FI (g)	3176.66	3133.87	3066.33	3181.62	34.69	0.91	0.45	0.65
FCR	1.79^a^	1.65^b^	1.65 ^b^	1.61^b^	0.01	0.004	0.13	0.25
PER	2.68^b^	2.91^ab^	2.92^ab^	2.98^a^	0.02	0.006	0.17	0.28
RGR	184.21^b^	185.03^ab^	184.69^ab^	185.50^a^	0.13	0.02	0.99	0.09

^a,b^ Means within the same row carrying different superscripts are significantly different (*P* < 0.05). BW: body weight, BWG: body weight gain, FI: feed intake, FCR: feed conversion ratio, PER: protein efficiency ratio, RGR: relative growth rate. T1: Control group; T2, T3, and T4: basal diet supplemented with 0.15 or 0.45 or 0.75% mixture of GLM and EO, respectively. * Regression is significant at *P *< 0.05

### Ileal amino acids digestibility

Table 2 shows the AID%. Compared to the control, dietary GLM and oregano EO at 0.45% quadratically decreased (*P* < 0.05) the AID% of threonine; however, a quadratic and cubic decrease (*P* < 0.05) in the AID% of arginine observed at 0.15% dietary GLM and oregano EO. The AID% of lysine, methionine, tryptophan, valine, leucine, and isoleucine did not show significant (*P >* 0.05) changes between all experimental treatments.

**Table 2:** Effects of supplemental GLM and oregano EO blend on the broilers’ apparent ileal digestibility coefficient (AID%) of amino acids

Parameters	T1	T2	T3	T4	SEM	Regression *		
						Linear	Quadratic	Cubic
Lysine	89.12	89.38	88.95	88.95	0.07	0.17	0.37	0.11
Methionine	87.54	87.57	87.7	87.08	0.05	0.25	0.19	0.41
Threonine	85.71^a^	84.81^ab^	84.35^b^	85.67^a^	0.11	0.53	0.001	0.17
Tryptophan	87.67	88.28	87.90	87.22	0.17	0.40	0.18	0.73
Arginine	90.45^a^	90.04^b^	90.28^ab^	90.31^ab^	0.04	0.62	0.02	0.03
Valine	86.01	85.87	85.69	85.76	0.06	0.20	0.49	0.68
Leucine	90.68	90.56	90.74	90.67	0.05	0.82	0.82	0.33
Isoleucine	86.10	86.10	86.16	86.26	0.04	0.39	0.71	0.97

^a,b^ Means within the same row carrying different superscripts are significantly different (*P* < 0.05). T1: Control group; T2, T3, and T4: basal diet supplemented with 0.15 or 0.45 or 0.75% mixture of GLM and EO, respectively. * Regression is significant at *P* < 0.05

### Histomorphology of the small intestine

**Fig. 2:**
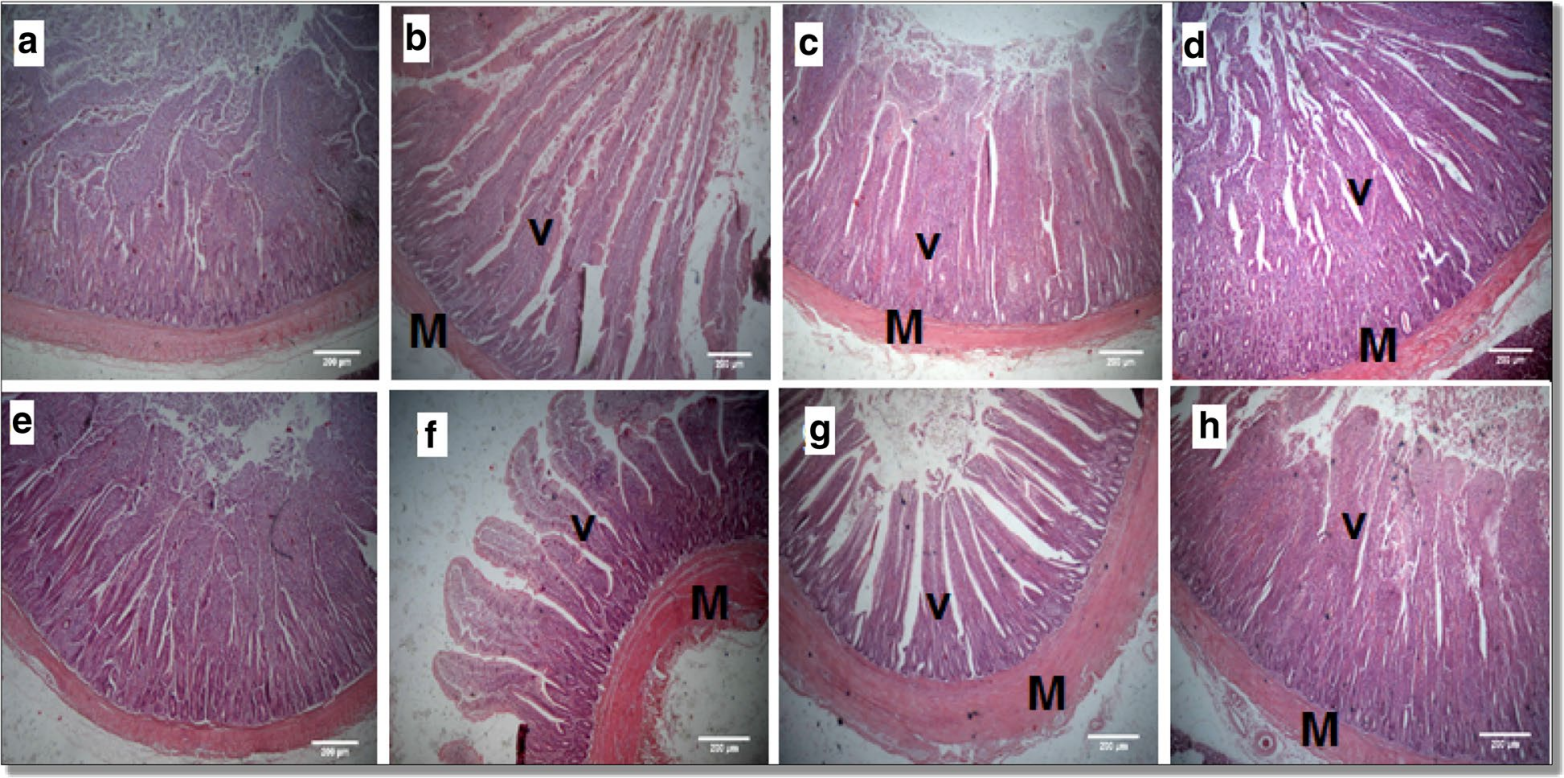
A representative photomicrograph of H&E stained small intestine sections of the broiler chickens. Duodenal sections (**b**, **c**, **d**) from T2, T3 and T4 respectively, showing increased villus (V) height in GLM and oregano EO supplemented groups compared to the control group (**a**). Jejunal section (f, g, h) from T2, T3, and T4, respectively, showing an increase in villus (V) height and muscle (M) thickness in GLM and oregano EO supplemented compared to the control group (**e**). T2, T3, and T4: basal diet supplemented with 0.15 or 0.45 or 0.75% mixture of GLM and EO, respectively

**Fig. 3:**
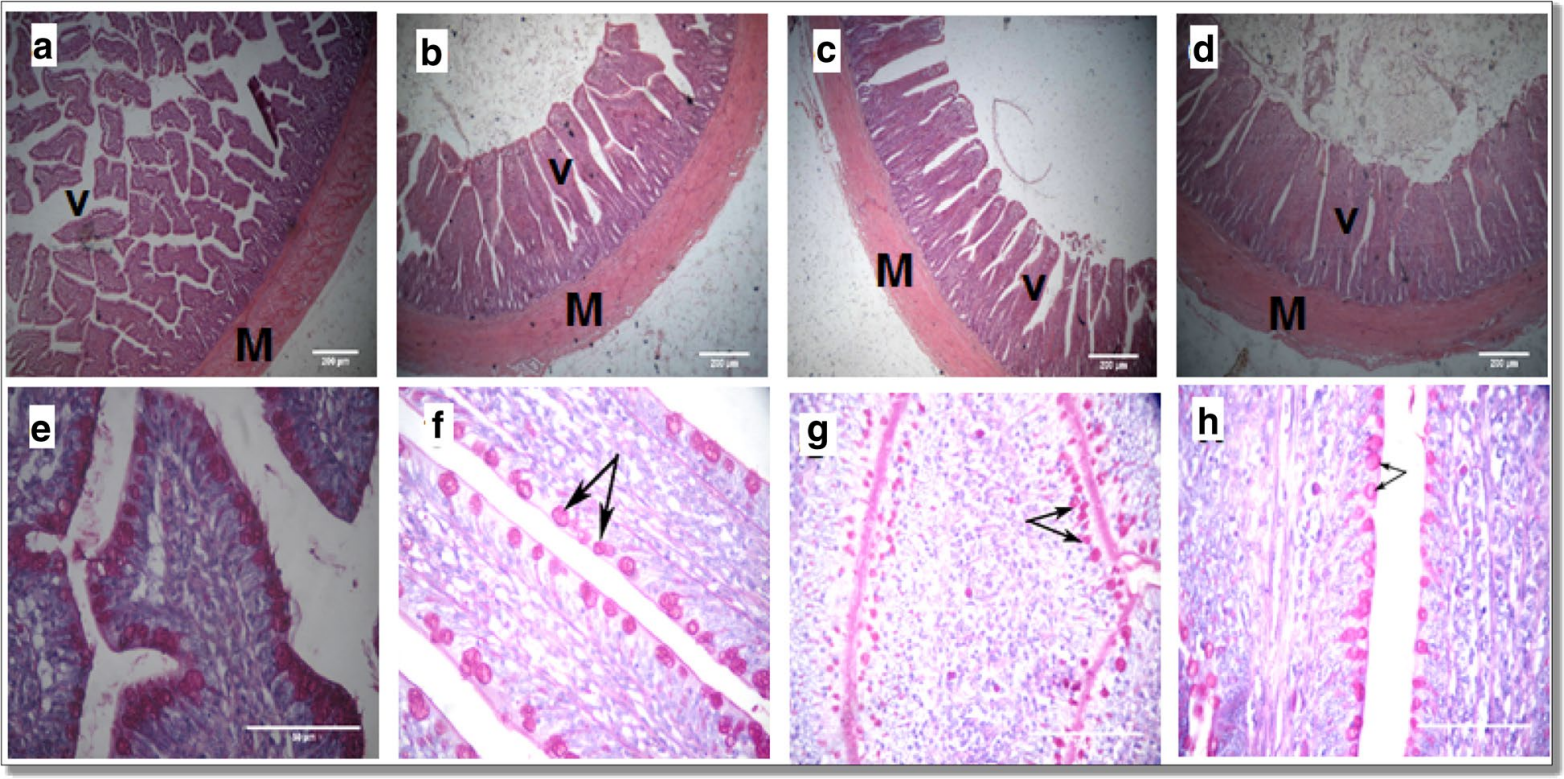
A representative photomicrograph of H&E stained small intestine sections of the broiler chickens. Ileal sections (**b**, **c**, **d**) from T2, T3, and T4, respectively, showed an increase in the villus (V) height and increased muscle (M) thickness in GLM and oregano EO supplemented groups compared to the control group (**a**). Goblet cells (arrows) in the PAS-stained ileum (**f**, **g**, **h**) from T2, T3, and T4, respectively, showed decreased goblet cells count in all GLM and oregano EO supplemented groups compared to the control (**e**). T2, T3, and T4: basal diet supplemented with 0.15 or 0.45 or 0.75% mixture of GLM and EO, respectively.

The histomorphology of small intestine sections is shown in Table 3, Figs. 2 and 3. A linear and quadratic increase in the duodenal muscle thickness was found at 0.45 and 0.75% dietary GLM and oregano EO blend (*P* < 0.01). A linear and quadratic increase was observed in the duodenal crypt depth at 0.75% dietary supplement (*P* < 0.01) with a decrease recorded in the duodenal crypt depth at 0.15% dietary level (*P* < 0.05). Compared to the control, all GLM and oregano EO supplemented groups showed a linear, quadratic, and cubic increase in duodenal and ileal villus height and ileal muscle thickness (*P* < 0.01). The duodenal goblet cell count was linearly decreased by the supplement (*P* = 0.02). Compared to the control group, linear and quadratic increases in the jejunal muscle thickness; linear and cubic increases in the jejunal villous height were observed in all GLM and oregano EO supplemented groups (*P* < 0.01). No change (*P >* 0.05) was shown in jejunal crypt depth among all experimental groups. A linear decrease in the jejunal goblet cell count was found at 0.45 and 0.75% dietary GLM and oregano EO blend (*P* < 0.01). A quadratic and cubic increase in the ileal crypt depth was observed at 0.15% dietary supplement (*P* < 0.01). A linear and quadratic decrease in the ileal goblet cell count was observed in all GLM and oregano EO supplemented groups (*P* < 0.01).

**Table 3:** Effects of supplemental GLM and oregano EO blend on the broilers’ morphometric measures (μm) of the small intestine

Parameters	T1	T2	T3	T4	SEM	Regression *		
						Linear	Quadratic	Cubic
Duodenum								
Muscle thickness	121.80^c^	114.23^c^	168.8^b^	229.10^a^	10.30	0.00	0.00	0.05
Crypt depth	247.98^b^	158.22^c^	236.41^b^	345.98^a^	11.78	0.00	0.00	0.06
Villus height	582.39^b^	1213.89^a^	1185.71^a^	1192.57^a^	50.01	0.00	0.00	0.002
Goblet cell count	25^a^	18.33^b^	20^b^	17.66^b^	1.22	0.02	0.22	0.13
Jejunum								
Muscle thickness	117.06^c^	387.91^a^	416.42^a^	237.89^b^	21.90	0.001	0.00	0.66
Crypt depth	252.83	278.4	216.46	222.06	10.60	0.05	0.54	0.05
Villus height	672.81^c^	1070.14^b^	962.60^b^	1251.40^a^	55.57	0.00	0.09	0.00
Goblet cell count	40.66^a^	32.33^ab^	27.66^b^	25^b^	1.48	0.00	0.16	0.84
Ileum								
Muscle thickness	219.02^b^	356.87^a^	336.22^a^	366.26^a^	15.93	0.00	0.00	0.001
Crypt depth	117.06^b^	209.70^a^	133.64^b^	138.99^b^	8.59	0.81	0.002	0.00
Villus height	323.87^c^	619.15^ab^	562.82^b^	634.54^a^	23.81	0.00	0.00	0.00
Goblet cell count	70^a^	55^b^	45.33^b^	52.66^b^	2.37	0.001	0.004	0.37

^a,b,c ^Means within the same row carrying different superscripts are significantly different (*P <* 0.05).T1: Control group; T2, T3, and T4: basal diet supplemented with 0.15 or 0.45 or 0.75% mixture of GLM and EO, respectively. * Regression is significant at *P* < 0.05

### Blood biochemical parameters and immune status

Data regarding the effect of GLM and oregano EO blend on the biochemical parameters are shown in Table 4. The GLM and oregano EO supplemented groups did not show significant (*P >* 0.05) changes in serum TC, TG, HDL-C, and LDL-C levels. There was a linear increase in the ALP level by the supplement (*P* = 0.03). All GLM and oregano EO dietary levels did not result in significant (*P >* 0.05) changes in serum IgM, IL10, or C3.

**Table 4:** Effects of supplemental GLM and oregano EO blend on the broilers’ blood biochemical parameters and immune status:

Parameters	T1	T2	T3	T4	SEM	Regression *		
						Linear	Quadratic	Cubic
TC (mg/dl)	223.78	238.98	220.98	216.33	2.29	0.18	0.15	0.11
TG (mg/dl)	100.03	90.37	147.07	158.01	5.73	0.03	0.63	0.62
HDL(mg/dl)	53.95	55.17	60.30	48.32	3.54	0.68	0.31	0.45
LDL(mg/dl)	153.66	160.23	170.29	183.02	8.65	0.23	0.86	0.99
ALP (U/L)	35.99^b^	46.34^ab^	53.73^ab^	72.36^a^	6.75	0.03	0.70	0.76
IgM (mg/dl)	110.66	92.95	86.46	62.90	9.89	0.05	0.84	0.66
C3 (mg/dl)	74.66	80.74	100.45	100.11	4.55	0.26	0.85	0.66
IL10 (pg/ml)	0.12	0.15	0.17	0.13	0.01	0.71	0.15	0.67

^a, b ^Means within the same row carrying different superscripts are significantly different at (*P* < 0.05). TC: total cholesterol, TG: triglycerides, HDL: high-density lipoprotein, LDL: Low-density lipoprotein, C3: Complement 3, IL10: interleukin 10. T1: Control group; T2, T3, and T4: basal diet supplemented with 0.15 or 0.45 or 0.75% mixture of GLM and EO, respectively. * Regression is significant at *P* < 0.05

### Economic efficiency

As shown in Table 5, there was a linear increase in the total return and performance index at 0.75% dietary level (*P* < 0.05). In contrast, the feed costs, total costs, net profit, feed cost/kg gain, and economic efficiency remained unaffected by the supplementation.

**Table 5:** Effects of supplemental GLM and oregano EO blend on the economic value of the diets

Parameters	T1	T2	T3	T4	SEM	Regression *		
						Linear	Quadratic	Cubic
Total return (USD)/bird	2.69^b^	2.88^ab^	2.82^ab^	2.98^a^	0.043	0.04	0.87	0.17
Net profit (USD)	0.92	1.10	1.02	1.08	0.027	0.09	0.26	0.08
Total costs (USD)	1.76	1.77	1.79	1.89	0.024	0.06	0.32	0.74
Feed costs (USD)	1.28	1.29	1.32	1.42	0.024	0.06	0.32	0.74
Economic efficiency	0.71	0.85	0.77	0.76	0.020	0.68	0.08	0.12
Feed cost/kg gain (USD)	0.72	0.68	0.71	0.72	0.007	0.97	0.07	0.18
Performance index%	92.34^b^	105.31^ab^	104.47^ab^	114.25^a^	2.71	0.007	0.71	0.21

^a, b^ Means within the same row carrying different superscripts are significantly different at (P < 0.05). T1: Control group; T2, T3, and T4: basal diet supplemented with 0.15 or 0.45 or 0.75% mixture of GLM and EO, respectively. * Regression is significant at *P* < 0.05

## Discussion

Our study aimed to determine if GLM and oregano EO's combined inclusion to broilers’ diets as natural alternatives to antibiotics and chemical feed additives would boost chickens’ performance and general health. As known, antibiotics use in chicken diets has been linked to many negative consequences, including antibiotic residue accumulation on meat and the development of antibiotic-resistant microorganisms [17]. Furthermore, worldwide antibiotic use in animal production results in the release of enormous amounts of antibiotics into the environment, which continues the bioaccumulation of antibiotics in the environment, leading to environmental contamination [18]. As a result, there is a rising concern about the risks that antibiotics used in poultry production bring to human health, which has prompted research into natural alternatives. In this study, the improvement in broilers growth performance in response to dietary GLM and oregano EO was recorded in the term of improved FCR from day 11 onward, with the highest dietary level of GLM and oregano EO showing the most improvement during the finisher stage. The overall performance showed a linear increase in the BW, BWG, PER, and RGR and a linear decrease in the FCR at 0.75% dietary level of GLM and oregano EO compared to the control and the optimum dietary level of GLM and oregano EO blend detected using broken-line regression model was 0.58% based on the data of final BW and FCR. The improved growth can be attributed to the improvement of most intestinal morphometrics, which leads to an increase in the absorption surface available for the absorption of nutrients essential for growth [9, 19]. However, dietary GLM and oregano EO blend had an insignificant impact on the AID% of most identified essential AA, with significant declines in AID% of threonine and arginine at different dietary levels of supplemented additives. These AAs control the critical metabolic pathways that regulate organisms' overall growth [20]. Indeed, AA has been shown to control the expression of biomarkers required for proper gastrointestinal tract function, particularly in threonine and arginine, which have been investigated for this function [21]. Aside from its role as a component of intestinal mucin, threonine catabolism generates various metabolically essential metabolites such as glycine, pyruvate, and acetyl-CoA [22, 23]. Likewise, arginine is identified for its role as a precursor for the production of polyamines, nitric oxide, and creatine and its ability to promote insulin growth factor release, which has a variety of biological roles [24]. We previously demonstrated that broilers fed graded levels of GLM had no improvement in growth performance traits [9], suggesting that combined supplementation of GLM with oregano EO could increase chicken growth performance moderately. This improvement may be due to the presence of EO, which provides active components of antimicrobial, antifungal, and antioxidant properties, which all positively impact growth performance in terms of FCR and PER [25]. These active components have been shown to allow birds to use dietary nutrients more efficiently [26, 27], which contradicts the existing AID% findings. As a result, further research into the effects of dietary GLM and oregano EO on chicken growth and nutrient utilization still required.

The combined supplementation of GLM and oregano EO at different levels in broiler diets led to an intestinal improvement in terms of muscle thickness, villus height in the assayed parts of the small intestine. Nevertheless, duodenal crypts showed decreased depth at the lowest dietary level of GLM and oregano EO. In broilers, the small intestine, primarily the duodenum and jejunum, plays a central role in feed nutrients digestion, and absorption. Better small intestinal development leads to enhanced nutrient use, directly correlates with growth rate [28]. Such intestinal development mostly assessed using villus length, crypt depth, and muscle thickness, with longer villus, lower crypt depth, and thicker muscle leading to higher mucosal surface area and greater digestive efficiency in broilers [29]. In our earlier study, Amer *et al.* [9] demonstrated improved intestinal histomorphology by GML supplementation except for the ileum suggesting that the improved ileal morphometric measures in the current study may be due to oregano EO addition. Intriguingly, the observed increase in intestinal muscle thickness and villus height in the present study did not translate to improved nutrient utilization in the chickens (i.e., AID%).

Furthermore, the goblet cell count is used to evaluate the condition of the small intestine in broilers. We found a significant decrease or no change in goblet cell counts in response to dietary GLM and oregano EO compared to the control group. Goblet cells are located in the epithelial layer all along the intestinal villi, where they secrete mucin glycoproteins, which are part of the mucus layer that protects the intestinal surface from environmental toxins, microorganisms, and specific coarse dietary components [30]. There is no clarity on whether a rise in goblet cell number and area is an indication of better bird health; however, the mucin production in the gastrointestinal tract, which is needed for various brush borders processes, depends on such cell numbers and areas [31]. This suggests that supplemental GLM and oregano EO has no impact on the mucin profile in the small intestine of broilers. As far as we know, there is a lack of research investigating the effect of a blend of MCFAs with phytogenic feed additives on chickens' intestinal morphology; however, earlier research recorded an improvement in chickens’ intestinal health in response to dietary MCFAs [32, 33]. Previous research on the effect of short-chain fatty acids (SCFAs) on broiler gut health by Pan and Yu [34] attributed the improvement in intestinal health to the SCFAs' involvement as an energy source for intestinal epithelial cells, which in turn enhances their growth and proliferation diet [3–5]. In the same context, the current improvement in intestinal morphology (i.e., muscle thickness and villus height) because of dietary MCFAs could be owed to the same notion of Pan and Yu [34], particularly the medium-chain triglycerides derived from MCFAs are reported to be easily absorbed by enterocytes in the intestine [35].

Meanwhile, chickens’ metabolic response in our experiment did not show significant changes except for that of ALP enzymatic activity. We reported no change in serum lipid profile in response to dietary GLM and oregano EO compared to the control with a linear increase in the ALP enzymatic activity. To the best of our knowledge, there has been little research into the effect of MCFAs and EO blends on broilers' lipid profile or enzymatic activity. The study by Omar *et al.* [10] showed a lowered lipid profile (i.e., total lipids, TGs, and TC) in broilers fed with the combined supplementation of thyme oil (0.1 and 0.2 g/kg diet) and MCFAs (1 g/kg diet), which disagree with our results. Such disagreement may be due to the different types and dosages of EO and MCFAs used.

On the other hand, earlier research investigated the impacts of dietary MCFAs or EO as separate supplements [9, 36, 37]. Liu *et al.* [36] found no change in TC and LDL-C serum levels in broilers in response to various medium-chain œ-monoglycerides dietary levels, but a rise in HDL-C level was observed. Also, in a previous study, we found that adding GLM to broiler diets raised HDL-C levels while decreased TG levels at 1 and 3 g kg ^–^ 1[9].

Furthermore, when rosemary, thyme, and oregano EOs were added in combination in broiler diets, an increase in HDL-C was observed with no difference in TGs and TC levels [37]. Such lipid profile results reported in previous studies may suggest an increase in lipid profile response to MCFAs or EOs when supplemented separately, despite combined supplementation as in our current study. Meanwhile, in our earlier study, we reported no impact of dietary GML on the enzymatic activity level of ALP when supplemented alone [9]; thus, based on the current findings of increasing ALP level, it seems that the combination of GLM and oregano EO may have a less protective effect on broiler health status [38]

No significant changes were detected in the assessed immune status indices (IgM, IL10, or C3). Although various studies have been conducted with MCFAs and EOs in poultry diets, their effect on birds' immune status is limited. Najafi and Taherpour [39] have reported enhancing broilers' immune status in terms of improved lymphocyte ratio with herbal oils supplementation, which could be attributed to the rising blood immunoglobulin levels and capacity of leukocytes to kill microbial cells due to terpinolene. Likewise, in a mice study, MCFAs were found to reduce the number of leukocytes, tumor necrosis factor, and IL6 levels in adipose tissue and total poly-morphonuclear circulating cells as a mechanism to reduce the inflammatory response [40]. In the previous study conducted by Amer *et al.* [9], increased serum IgM and IL10 were recorded in broilers due to 5 g GLM kg –1. Concerning earlier reports, it has been observed that supplemented GLM and oregano EO had no synergistic impact in our study, as shown by the lack of significant changes in the birds’ immune status.

A linear rise in the total return and performance index at the 0.75 % dietary level of supplemented additive accompanied the enhanced growth performance in the current study. These findings suggest that the additives applied seemed to be economically feasible. However, the market price of other feed ingredients at the time of feed formulation determines this [41].

## Conclusions

Based on the obtained findings, better growth performance was achieved at a 0.75 % dietary level of GLM and oregano EO blend by improving most intestinal morphometric measures. The optimum dietary level detected using broken-line regression was 0.58%. The lack of influence of supplemented additives on chickens' immune and metabolic responses could indicate a lack of synergy between GLM and oregano EO. Our findings could provoke future research into the impact of different doses of single supplementation of GLM or oregano EO in broiler diets to compare with the combined supplementation effect.

## Methods

### Birds, diets, and management

All the protocols for the current experiment were approved by the Institutional Animal Care and Use Committee of Zagazig University (ZU-IACUC–2020). All experiments were performed following the ARRIVE guidelines. Hatchling unsexed Ross-308 broilers (*n* = 240) were purchased from a commercial hatchery (Dakahlia Poultry, Mansoura, Egypt) and given a 3-day adaptation period before beginning the trial (initial body weight was 76.62 g ± 0.50). Chicks were randomly assigned to four experimental treatments (6 replicates/treatment and ten chicks/replicate) in a completely randomized design. Birds were fed a corn-soybean meal basal diet supplemented with four levels, 0 (control group without additives), 0.15, 0.45, and 0.75%, of a blend of GLM and oregano EO (BERGIN^®^ Proviplus OC12, Bergophor Futtermittelfabrik Dr. Berger GmbH& Co.KG, Kronacher Str. 13, D-95326 Kulmbach).

The lighting system was held at 23:1 h light/dark for the first 3 days, next 20:4 h light/dark till the end of the study. The study lasted for 35 days with feed in a mash form, and freshwater was provided *ad libitum* during that period. The starter diet was given to the chicks till 10 d of age, followed by the grower and finisher from day 11 to 22 and 23 to 35, respectively, according to the Ross-308 broilers feed specifications [42] (Table 6). After the study ended, all remaining chickens were released.

**Table 6:** Composition of the basal diet as fed basis (%)

Ingredients	Starter (4-10 d)	Grower (11-22 d)	Finisher (23-35 d)
Corn 7.5%	53.02	58.00	65.20
Soybean meal 47%	37.00	32.00	27.00
Corn gluten meal 60%	3.5	3.2	2.50
Oil (soya) - e76	2.00	3.00	1.80
Dicalcium phosphate DCP 18%	2.00	1.7	1.45
Calcium carbonate	0.7	0.5	0.50
Sodium bicarbonate	0.35	0.33	0.31
Dl methionine 99%	0.36	0.28	0.30
Broiler premix*	0.3	0.3	0.30
L-LYSINE HCL 98%	0.3	0.3	0.26
Salt	0.15	0.11	0.13
Antimycotoxin	0.1	0.1	0.10
Choline 60 veg	0.07	0.07	0.07
L-THREONINE 98.5%	0.1	0.1	0.07
Enzyme Phytase	0.05	0.01	0.01
Chemical analysis			
ME (Kcal/kg)	3,007.24	3,103.42	3,202.13
Moisture %	11.27	11.23	11.17
Crude protein %	23.70	21.55	20.13
Crude Fat %	4.98	6.04	6.68
Crude fiber %	3.426	3.23	2.98
Ash %	6.35	5.53	5.01
Av. Phosphorus (g/kg)	4.96	4.46	4.02
Calcium (g/kg)	9.47	8.95	8.21
Methionine (g/kg)	7.24	6.17	6.13
Lysine (g/kg)	14.55	13.21	11.58
Tryptophan (g/kg)	2.79	2.49	2.19
Threonine (g/kg)	9.98	9.12	8.11
Valine (g/kg)	11.32	10.27	9.58
Arginine (g/kg)	15.31	13.71	12.14
Sodium (g/kg)	1.85	1.62	1.67
Cl (g/kg)	2.62	2.31	2.34
Potassium (g/kg)	8.84	8.05	7.06

*Premix per kg of diet: vitamin D3, 200 IU; vitamin A, 1 500 IU; vitamin K3, 0.5 mg; vitamin E, 10 mg; riboflavin, 3.6 mg; thiamine, 1.8 mg; pyridoxine, 3.5 mg; folic acid, 0.55 mg; pantothenic acid, 10 mg; cobalamin, 0.01 mg; niacin, 35 mg; biotin, 0.15 mg; Cu, 8 mg; Fe, 80 mg; Mn, 60 mg; I, 0.35 mg; Zn, 40 mg; Se, 0.15 mg

### Growth performance

On the fourth day of age, the average initial body weight (BW) was recorded. The BW was then determined on a replicate basis at 10, 23, and 35 days and body weight gain (BWG) was consequently determined [9]. The difference between the weight of the provided feed and the feed that remained was used to calculate feed intake (FI) per replicate. Afterward, the relative growth rate (RGR) and feed conversion ratio (FCR) were estimated. FCR = quantity of consumed feed (g) / BWG (g) and RGR = $\left[\left(\mathrm{W}2-\mathrm{W}1\right)/\frac{\mathrm{W}1+\mathrm{W}2}{2}\right]\times 100$ where W1 and W2 are initial and final BW, respectively [43]. The protein efficiency ratio (PER) was calculated according to [44] as PER = Live BWG (g)/Protein intake (g).

### Ileal amino acids digestibility

The AA ileal digestibility was determined using titanium dioxide, an indigestible indicator material, as defined previously [45]. The AA content in the ileal digesta samples and diet were measured according to Li *et al*. [46]. Tryptophan was assessed separately [47], and then the method of Fenton and Fenton was used to value titanium dioxide [48]. The apparent ileal digestibility coefficient (AID%) was calculated as described in our earlier study [9].

### Sample collection and biochemical analyses

At the end of the study (35 days) and following a 12 h fasting period, 6 birds were taken randomly from each group for sample selection, and blood was collected into sterilized tubes without coagulant after euthanization using cervical dislocation, according to the American Veterinary Medical Association guidelines [49]. After allowing samples to clot at room temperature, they were centrifuged at 3500 rpm for 15 min to extract serum. Serum samples were then held in Eppendorf tubes and preserved at –20°C until further analysis. For histological examinations, samples were taken from various portions of the small intestine (duodenum, jejunum, ileum).

Total cholesterol (TC), triglycerides (TGs), high-density lipoprotein cholesterol (HDL-C), and low-density lipoprotein cholesterol (LDL-C) were determined in serum according to [50–52], respectively using commercial diagnostic kits (Egyptian Company for Biotechnology, Cairo, Egypt) as described in [9]. Serum alkaline phosphatase (ALP), interleukin 10 (IL10), and immunoglobulin M (IgM) were determined using chicken ELISA kits (MyBioSource Co. CAT.NO. MBS012469, MBS701683, and ABCAM Co. CAT.NO. AB157691, respectively). According to the manufacturer's guidelines, serum complement 3 (C3) level was determined using an ELISA kit (Life Span Biosciences, Inc.) with the code CAT.NO.LS-F9287.

### Histomorphological examination of the small intestine

At the end of the experiment, five birds/ group were euthanized using cervical dislocation, according to the American Veterinary Medical Association guidelines [49], and around 2 cm tissue samples were dissected out from different portions of the small intestine (duodenum, Jejunum, and ileum) following the earlier procedure [53]. The samples were kept for 72 h in 10% neutral buffered formaldehyde, then dehydrated and cleared before being embedded in wax. Two sections (2 cm long) were collected from each intestinal segment. Periodic acid-Schiff staining on 5-m thick transverse sections (sliced by a microtome), fixed on slides and stained with hematoxylin and eosin identified the acidic mucus-containing goblet cells. A digital camera (Canon) attached to a Zeiss light microscope was used to examine the mucosal and muscular layer of the intestinal parts (three fields/ section). Villus height, crypt depth, and thickness of the tunica muscularis were then determined [9]. The number of goblet cells/ unit of epithelial area (mm^2^) and single goblet cell areas (μm^2^) in PAS-stained sections was calculated using ImageJ software (National Institute of Health, USA).

### Economic efficiency

Collective efficiency measures, including total return, total costs, variable costs, and net profit, were calculated according to [54, 55].

$$\mathrm{Total}\ \mathrm{feed}\ \mathrm{cost}\ \left(\mathrm{USD}/\mathrm{bird}\right)=\mathrm{Total}\ \mathrm{feed}\ \mathrm{intake}/\mathrm{bird}\ \mathrm{x}\ \mathrm{Price}\ \mathrm{of}\ \mathrm{one}\ \mathrm{kg}\ \mathrm{feed}\ \mathrm{including}\ \mathrm{the}\ \mathrm{supplement}\ \left(5.57\ \mathrm{USD}/\mathrm{Kg}\ \mathrm{supplement}\right).$$

Total cost (USD/bird) was computed by considering feed cost and the expenses of one-day-old chick, litter, labor, veterinary services, electricity, and other miscellaneous expenditure, that were common to all groups.

$$\mathrm{Total}\ \mathrm{return}\ \left(\mathrm{USD}/\mathrm{bird}\right)=\mathrm{Live}\ \mathrm{body}\ \mathrm{weight}/\mathrm{bird}\ \mathrm{x}\ \mathrm{Price}\ \mathrm{of}\ \mathrm{kg}\ \mathrm{body}\ \mathrm{weight}.$$

$$\mathrm{Net}\ \mathrm{profit}\ \left(\mathrm{USD}/\mathrm{bird}\right)=\mathrm{Total}\ \mathrm{returns}-\mathrm{Total}\ \mathrm{costs}.$$

$$\mathrm{Economic}\ \mathrm{efficiency}\ \left(\mathrm{E}.\mathrm{EF}\right)=\mathrm{Net}\ \mathrm{profit}/\mathrm{Total}\ \mathrm{feed}\ \mathrm{cost}.$$

$$\mathrm{Feed}\ \mathrm{cost}/\mathrm{kg}\ \mathrm{gain}\ \left(\mathrm{USD}/\mathrm{bird}\right)=\mathrm{Total}\ \mathrm{feed}\ \mathrm{cost}/\mathrm{Total}\ \mathrm{weight}\ \mathrm{gain}.$$

The performance index (PI) was calculated based on a previous study [56].

$$\mathrm{Performance}\ \mathrm{index}\%\left(\mathrm{PI}\right)=\mathrm{final}\ \mathrm{live}\ \mathrm{body}\ \mathrm{weight}\ \left(\mathrm{kg}\right)/\mathrm{feed}\ \mathrm{conversion}\ \mathrm{x}\ 100.$$

### Statistical analysis

ANOVA test was applied based on polynomial orthogonal contrasts. Linear, quadratic, cubic regression equations were calculated using SPSS Version 17 for Windows (SPSS Inc., Chicago, Illinois, USA) after Shapiro-Wilk's test was used to verify the normality, and Levene’s test was used to verify homogeneity of variance components between experimental treatments. The replicate (*n* = 6) has been considered as an experimental unit. The significant differences between mean values were examined through Tukey's honestly significant difference test. For all the analyses, pooled SEs were established, and the degree of significance was set at *P* < 0.05. The broken-line regression with Tukey's test was considering data on BW and FCR for determining the optimum supplementation level of GML and EO blend [57].

## Data Availability

The datasets used and analyzed during the current study available from the corresponding author on reasonable request.

## Abbreviations

GLM: Glycerol monolaurate
EO: Essential oil
AID%: Amino acid ileal digestibility coefficient
BW: Body weight
BWG: Body weight gain
FI: Feed intake
FCR: Feed conversion ratio
RGR: Relative growth rate
PER: Protein efficiency ratio
Ti: Titanium dioxide
AA: Amino acid
